# Influence of antipsychotic medications on hyperlipidemia risk in patients with schizophrenia: evidence from a population-based cohort study and *in vitro* hepatic lipid homeostasis gene expression

**DOI:** 10.3389/fmed.2023.1137977

**Published:** 2023-06-22

**Authors:** Tien-Yuan Wu, Ni Tien, Cheng-Li Lin, Yu-Cun Cheah, Chung Y. Hsu, Fuu-Jen Tsai, Yi-Jen Fang, Yun-Ping Lim

**Affiliations:** ^1^Graduate Institute of Clinical Pharmacy, College of Medicine, Tzu Chi University, Hualien, Taiwan; ^2^Department of Pharmacy, Taichung Tzu Chi Hospital, Buddhist Tzu Chi Medical Foundation, Taichung, Taiwan; ^3^Department of Laboratory Medicine, China Medical University Hospital, Taichung, Taiwan; ^4^Department of Medical Laboratory Science and Biotechnology, China Medical University, Taichung, Taiwan; ^5^Management Office for Health Data, China Medical University Hospital, Taichung, Taiwan; ^6^Department of Pharmacy, College of Pharmacy, China Medical University, Taichung, Taiwan; ^7^Graduate Institute of Biomedical Sciences, China Medical University, Taichung, Taiwan; ^8^School of Chinese Medicine, College of Chinese Medicine, China Medical University, Taichung, Taiwan; ^9^Department of Medical Research, China Medical University Hospital, Taichung, Taiwan; ^10^Division of Medical Genetics, China Medical University Children's Hospital, Taichung, Taiwan; ^11^Department of Biotechnology and Bioinformatics, Asia University, Taichung, Taiwan; ^12^Research Center for Environmental Medicine, Kaohsiung Medical University, Kaohsiung, Taiwan; ^13^Ph.D. Program in Environmental and Occupational Medicine, College of Medicine, Kaohsiung Medical University and National Health Research Institutes, Kaohsiung, Taiwan; ^14^Department of Environmental Health, Graduate Institute of Clinical Medicine, Kaohsiung Medical University, Kaohsiung, Taiwan; ^15^Department of Post-Baccalaureate Medicine, College of Medicine, National Chung-Hsing University, Taichung, Taiwan; ^16^Digestive Disease Center, Show Chwan Memorial Hospital, Changhua, Taiwan; ^17^Department of Internal Medicine, China Medical University Hospital, Taichung, Taiwan

**Keywords:** schizophrenia, antipsychotic medications, hyperlipidemia, cohort study, lipid homeostasis

## Abstract

**Introduction:**

Schizophrenia increases the risk of mortality and cardiovascular disease (CVD) risk. However, the correlation between antipsychotics (APs) and CVD remains controversial. Hyperlipidemia is a significant risk factor for CVD.

**Methods:**

We conducted a nationwide population-based retrospective cohort study to investigate the effects of APs on the risk of hyperlipidemia and lipid homeostasis gene expression. We used data from the Longitudinal Health Insurance Database of Taiwan on new-onset schizophrenia patients and a comparison cohort without schizophrenia. We used a Cox proportional hazards regression model to analyze the differences in hyperlipidemia development between the two cohorts. Furthermore, we examined the effects of APs on the hepatic expression of lipid homeostasis-related genes.

**Results:**

After adjusting for potential interrelated confounding factors, the case group (*N* = 4,533) was found to have a higher hyperlipidemia risk than the control cohort (*N* = 4,533) [adjusted hazard ratio (aHR), 1.30, *p* < 0.001]. Patients with schizophrenia without APs had a significantly higher risk of hyperlipidemia (aHR, 2.16; *p* < 0.001). However, patients receiving APs had a significantly lower risk of hyperlipidemia than patients not receiving APs (all aHR ≤ 0.42, *p* < 0.001). First-generation antipsychotics (FGAs) induce the expression of hepatic lipid catabolism genes in an in vitro model.

**Discussion:**

Patients with schizophrenia had a higher risk of hyperlipidemia than controls; however, compared with non-treated patients, AP users had a lower risk of hyperlipidemia. Early diagnosis and management of hyperlipidemia may help prevent CVD.

## Introduction

Schizophrenia is a brain disorder characterized by the long-term persistence of multiple mental problems; however, the disease is treatable with a better prognosis in some cases (1). It affects approximately 1% of the global population (2). Symptoms may persist for 30–50% of their lifetime and impair social functioning participation (3). Compared to the general population, patients with schizophrenia have a 12-fold higher mortality risk for various reasons, including somatic comorbidities, unhealthy lifestyles, and increased suicide rates (3). Cardiovascular disease (CVD) risk was 1.80-fold [95% confidence interval (CI) 1.71–1.88], coronary heart disease (CHD) was 1.54-fold (95% CI 1.30–1.82), and lipid profile abnormalities were observed compared with the general population (2) even before initiating antipsychotic (APs) treatment (4). In addition to the high suicide rate, a key reason for high morbidity and mortality is the associated abnormal blood lipid profile and concomitant CVD with a 2- to 3-fold increased risk, which makes these patients susceptible to increased cardiac mortality (5). The identified risk factors for CVD include obesity, less physical activity, high calories diet, smoking, hypertension, high blood glucose, and hyperlipidemia [increased total cholesterol (TC, ≥ 240 mg/dL); low-density lipoprotein cholesterol (LDL-C, ≥ 160 mg/dL), decreased high-density lipoprotein cholesterol (HDL-C, < 40 mg/dL), and elevated triglycerides (TGs, ≥ 150 mg/dL)]. The CVD risk is lower in the first episode than in multi-episode and long-duration illnesses in patients with schizophrenia (3). Therefore, CVD management is required in these patients.

AP medication is the primary treatment based on the basic symptoms of schizophrenia. First-generation APs (FGAs or typical APs) are dopamine antagonists that block the neurotransmitter’s three primary routes. In contrast, second-generation APs (SGAs or atypical APs) are expected to be more selective for the mesolimbic system, as they exhibit affinity for both dopamine and 5-HT_2_ receptors (6). Because schizophrenia is a chronic illness that requires constant attention, AP adverse effects build up over time, and long-term treatment with APs may influence the health status of patients owing to their side effects, such as metabolic syndrome (MetS). Patients with schizophrenia have a higher prevalence of MetS than controls (20.1% vs. 5.5%) (1). MetS significantly correlates with lipid homeostasis disorders and increased CVD risk and morbidity. The association between schizophrenia and hyperlipidemia was assessed before AP treatment. Studies comparing the baseline blood lipids of drug-naïve, first-episode psychosis patients with those of the general population provide the best possible alternative. These studies found impaired lipid metabolism in drug-naïve patients; TC and LDL-C showed an increase, whereas HDL-C was unchanged compared to controls (7–12), although not all showed the same findings. These studies only offer a “cross-sectional” perspective on the potential link between the metabolic characteristics of people with schizophrenia who are only beginning to experience symptoms. However, they do not state that whether or not these individuals are exposed to APs, their real metabolic status would change several years after the commencement of their illness. These observations suggest that the disease state independently influences adverse metabolic symptoms beyond treatment-induced effects.

Although patients’ lifestyles or genetic backgrounds may contribute to the predisposed confounding factors toward metabolic conditions (13), APs may contribute to and play a significant role in modulating the metabolic pathway in these patients. A longitudinal study reported that the prevalence of MetS in drug-naïve patients ranged from 0 to 14% at baseline; however, it increased to 52.4% in 3 months after starting APs (1). According to a prior study, people with schizophrenia had a 2–3 times greater prevalence and incidence of MetS than the general population, and the use of APs did not quantify these risks (14, 15). Treatment with AP causes weight gain and metabolic dysregulation. The reason for this phenomenon is fairly complex, involving factors such as neurochemical and hormonal consequences, depending on the APs provided. An increase in hunger that leads to weight gain may explain the possible role of APs in metabolic problems. However, not all clinicians agree that this is a medication-associated adverse effect (16). APs may help treat these patients and reduce the remission of symptoms, which is traditionally achieved through the blockade of the dopamine 2 receptor. Because of their serotonin antagonism, specifically at 5-HT_2A_ and 5-HT_2C_, their potential link to alleviating negative symptoms, and their mitigation of extrapyramidal side effects, the first-line therapy for schizophrenia is currently SGAs (16).

A randomized, double-blind, flexible-dose, multisite investigation of individuals with early schizophrenia reported that the treatment-related risk of MetS is high with clozapine and olanzapine (which have the highest affinity for histamine receptors), which have a moderate effect; risperidone has a mild effect; and aripiprazole, haloperidol, perphenazine, and ziprasidone have a low effect. Among these drugs, clozapine, olanzapine, and quetiapine have been found to worsen dyslipidemia (17, 18). However, the reason for this drug-induced hyperlipidemia remains uncertain because of inconsistencies among these studies (19–22). Compared to SGAs, FGAs have also been associated with increased body weight and lipid profiles (23); however, studies have concluded that the high incidence of MetS in patients with schizophrenia could not be solely attributed to APs.

The liver plays a critical role in regulating lipid metabolism, including *de novo* lipogenesis and catabolism. Abnormalities in lipid metabolism can lead to fat buildup in the liver, which is closely related to the onset of hyperlipidemia, a significant feature of metabolic syndrome (MetS). LDL-receptor-related protein 1 (LRP1), a transmembrane protein expressed in hepatocytes, is a crucial component of lipid homeostasis (24). LRP1 binds and internalizes ligands such as apolipoprotein E (apoE), primarily degraded by lysosomes (24). Liver-specific LRP1 knockout mice have been shown to have increased liver steatosis and hyperlipidemia, indicating that LRP1 is essential for maintaining lipid homeostasis (25). Hepatic lipase (HL) is another key regulator of lipoprotein metabolism. HL hydrolyzes TGs and phospholipids found in circulating plasma lipoproteins and promotes their absorption by cell surface receptors and proteoglycans (26). By regulating lipoprotein metabolism, HL can significantly affect atherogenesis and CVD onset. Free fatty acids (FFAs) are activated by fatty acyl-CoA derivatives during hepatic fatty acid degradation (26). Mitochondria and peroxisomes absorb activated fatty acyl-CoAs for the FA oxidation-mediated breakdown of acetyl-CoA. Lipid metabolism is carried out by lipoprotein lipase (LPL) through the hydrolysis of TG in chylomicrons and VLDL into glycerol and FAs in circulation (27). Hepatic LPL hydrolyzes TG in the liver and suppresses hepatic lipid accumulation. Insulin induces LPL expression and activity, and peripheral organs take up free FFAs for energy storage and consumption. Overall, a complex interplay of proteins, enzymes, and receptors regulates lipid metabolism in the liver, which has significant implications for the onset of hyperlipidemia, MetS, and CVD.

This is important for routine physical health screening, particularly for monitoring blood lipid levels, in patients with abnormal mental health, especially those treated with APs. Recommendations made by the American Diabetes Association and the American Psychiatric Association in 2004 include monitoring weight, waist circumference, blood pressure, fasting plasma glucose levels, and fasting lipid profiles (16). However, in real-world practice, such monitoring is relatively infrequent. Patients with schizophrenia are not consistently followed up to prevent future CVD consequences from MetS owing to their sedentary lifestyle, negative symptoms, and vulnerability to stress, regardless of APs medications (28). However, not all patients treated with APs show an increased incidence of these risk factors.

The primary aim of this study was to assess the risk of hyperlipidemia in patients with schizophrenia and the effect of APs on this risk. The secondary purpose of this study was to determine the expression patterns of lipid catabolism genes and the influence of FGAs and SGAs on lipogenesis-related genes. No observational cohort study has examined hyperlipidemia rates in patients with and without APs, especially in the Asian population. Understanding these parameters would guide psychiatric clinicians in identifying patients at risk of CVD. Therefore, we conducted a retrospective cohort study focusing on the clinical relevance and possible action of these drugs on lipid homeostasis-related genes.

## Materials and methods

### Data source

The National Health Insurance Research Database (NHIRD) covers approximately 99% of the Taiwanese population and has gathered patient information since 1995. The NHIRD contains detailed medical records of outpatient visits or admission, diagnostic codes, and prescription medications. We studied the subset dataset of the NHIRD, the Longitudinal Health Insurance Database (LHID), which has two million beneficiaries, to investigate the association between schizophrenia and hyperlipidemia. The individual’s information in the NHIRD could not be identified. The International Classification of Diseases, Ninth and Tenth Revision, Clinical Modification (ICD-9-CM, ICD-10-CM) were used to classify diseases. The Institutional Review Board of China Medical University Hospital Research Ethics Committee [CMUH109-REC2-031(CR-2)] approved this study.

### Study population

Here, we evaluated the association between non-schizophrenia and schizophrenia in terms of the risk of hyperlipidemia from 2000 to 2017. The case group was identified as the first diagnosis date for schizophrenia, and within the study period, a random date served as the control group. We excluded individuals <20 years of age, those diagnosed with hyperlipidemia before the index date, those who died, or those who withdrew from the insurance program during the study period. The propensity score matched a case with one control for sex, age (5 years interval), and the index year.

### Main outcome and comorbidities

The primary endpoints of this study were newly diagnosed hyperlipidemia (ICD-9-CM code 272; ICD-10-CM code E78). We defined the end date as the date until individuals had been diagnosed with hyperlipidemia, withdrew from the NHIRD, died, or December 31, 2017. We analyzed the inpatient and outpatient files to determine the individuals’ comorbidity status during the study period. Our inclusion criteria were patients with at least two inpatient diagnoses or one outpatient diagnosis. The comorbidities included type 2 diabetes mellitus (T2DM, ICD-9-CM code 250; ICD-10-CM code E08-E13), sleep disorder (ICD-9-CM 327.23, 780.51, 780.53, and 780.57), coronary artery disease (CAD, ICD-9-CM code 410–414; ICD-10-CM code I20.0, I20.1, I20.8, I20.9, I21, I22, I24.1, I24.8, I24.9, I25.1, and I25.2), hypertension (ICD-9-CM code 401–405; ICD-10-CM code I10, I11.0, I11.9, I12.0, I12.9, I13.0, I13.10, I13.11, I15.0, I15.1, I15.8, and I15.9), chronic kidney disease (CKD, ICD-9-CM code 585, 586; ICD-10-CM code N18, N19). We selected schizophrenia-associated medications that were considered confounding factors, including FGAs and SGAs.

### Chemicals and cell culture

The main chemicals used in this study, including FGAs (chlorpromazine, chlorprothixene, clothiapine, droperidol, flupentixol, fluphenazine, haloperidol, levomepromazine, loxapine, pimozide, prochlorperazine, sulpiride, thioridazine, and trifluoperazine) and SGAs (amisulpride, aripiprazole, brexpiprazole, clozapine, lurasidone, olanzapine, paliperidone, quetiapine, risperidone, ziprasidone, and zotepine) were acquired from Sigma-Aldrich (St. Louis, Missouri, USA) and were of highest purity grade. Before usage, the compounds were dissolved in dimethyl sulfoxide (DMSO) at the proper concentrations. Human hepatoma HepaRG™ cells were purchased from Thermo Fisher Scientific (Waltham, Massachusetts, USA). For 2 weeks, frozen cells were defrosted and kept alive in Williams’ E medium (Sigma-Aldrich, St. Louis, Missouri, USA) with 10% FetalClone™ II serum (HycloneTM, GE Healthcare, Chicago, Illinois, USA), 1 × L-glutamine, 5 μg/mL human insulin, and 50 μM hydrocortisone hemisuccinate without antibiotics as supplements. To produce differentiated hepatocyte-like features, the medium was then changed to the aforementioned medium plus 2% DMSO for an additional 2 weeks. Cells were grown at 37°C in a humidified 5% CO_2_ environment. P-nitrophenylphosphate (PNPP) was used in an acid phosphatase (ACP) experiment to measure cell viability (29).

### RNA isolation and quantitative real-time PCR (qRT-PCR) analysis

Gene expression levels were assessed to evaluate how APs affected the expression of genes involved in hepatic lipid homeostasis. Using a Direct-zol™ RNA MiniPrep kit (ZYMO Research, Irvine, CA, USA), total RNA was extracted from differentiated HepaRG cells under varied treatment conditions in accordance with the manufacturer’s instructions. Calculating the ratio of absorbance at 260/280 nm (ratio ≥ 1.8) allowed researchers to verify the quantity and purity of the RNA. Using a MultiScribe™ reverse transcriptase kit, first-strand cDNA synthesis was performed on 1 μg of total RNA (Thermo Fisher Scientific, Waltham, MA, USA). Expression of *LRP1*, *HL*, *LPL*, *LXRα*, *SREBP-1c*, *FAS*, *SCD*, and *β-actin* was evaluated using a StepOnePlus™ Real-Time PCR System and a Luminaris Color HiGreen qPCR master mix (Thermo Fisher Scientific, Waltham, Massachusetts, USA) according to established protocols. Supplementary Table S1 lists each pair of primers utilized for the real-time PCR study. The fractional PCR threshold cycle (Ct) value was used to calculate the target cDNA concentration in each sample. The target cDNA expression was determined using the formula 2^−(Ct target gene – Ct *β-actin*)^, with the relative mRNA levels normalized to those of *β-actin*. The information is displayed as fold-changes from the control group, and the experiment was performed in triplicates.

### Statistical analysis

The baseline demographic data for categorical variables were presented as counts and percentages between the study cohorts. Continuous variables such as age were presented as mean ± standard deviation (SD). Chi-square and *t*-tests were used to compare differences in the distribution of categorical and continuous variables between the case and control groups. To explore the relationship between schizophrenia and subsequent hyperlipidemia, we performed Cox proportional hazard regression analyses to present crude hazard ratios (HRs), adjusted hazard ratios (aHRs), and 95% CIs using unavailable and multivariable Cox proportional hazard regression models. A *p* value less than 0.05 was regarded as statistically significant when using SAS version 9.4 (SAS Institute Inc., Cary, NC, USA). R software was used to plot cumulative incidence curves, and the Kaplan–Meier (KM) plot was used to evaluate cumulative incidence.

Data from distinct measurements are presented as mean ± standard error (SE) for *in vitro* investigations. The Least Significant Difference test for multiple comparisons was used to determine the p value for each experimental comparison after a variance analysis. As shown in the figures, all *p* values were calculated in relation to the vehicle control group. All statistical analyses were performed using IBM SPSS Statistics for Windows, version 20.0 (IBM Corp., Armonk, N.Y., USA). The cutoff for statistical significance was *p* < 0.05.

## Results

### Baseline characteristics: demographic and association findings

**Figure 1:**
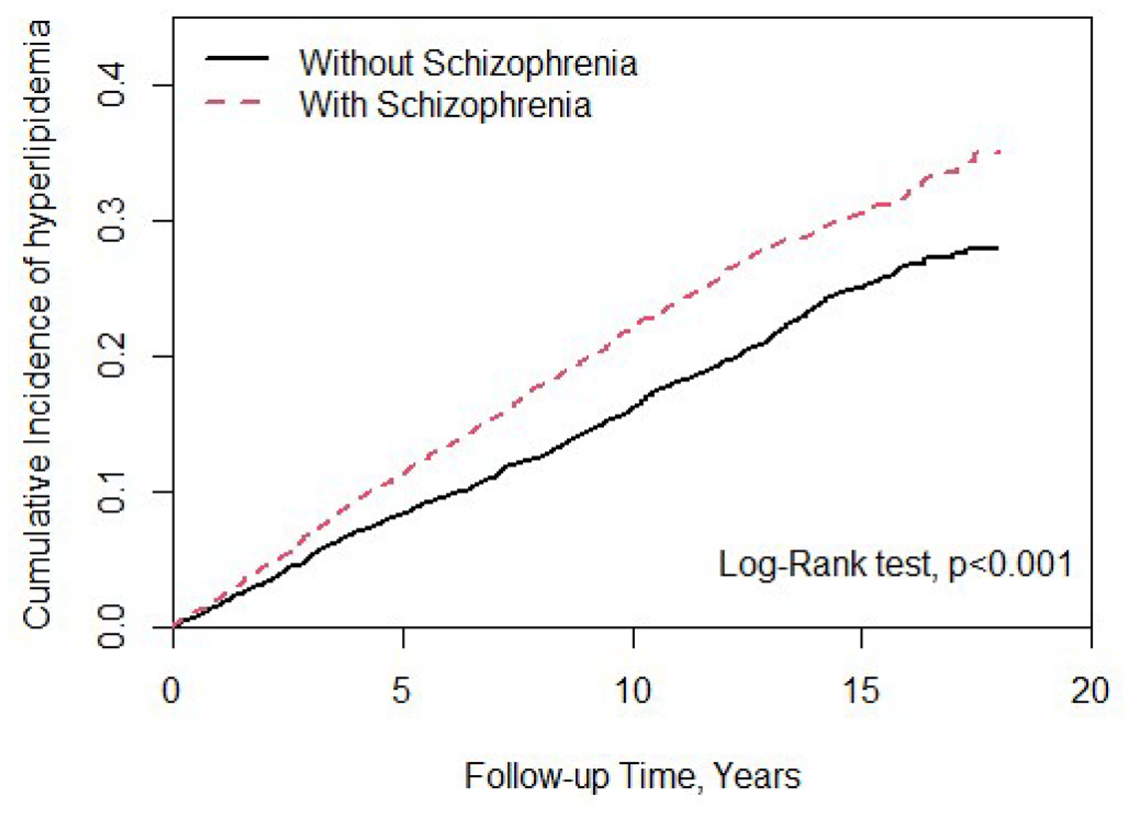
Cumulative incidence of hyperlipidemia compared between patients with and without schizophrenia using the Kaplan–Meier method. Case group mean follow-up years 9.93 (SD = 4.87). Control group mean follow-up years 9.12 (SD = 4.98).

There were 9,066 individuals in this cohort after propensity score matching by sex, age, index year, and comorbidities; we allocated 4,533 patients with schizophrenia to the case group and 4,533 individuals to the control group as non-schizophrenia. As shown in Table 1, 52.2% of participants in both case and control groups were male, and the mean ages in these groups were 38.0 (SD = 12.4) and 37.7 (SD = 13.0) years, respectively. In terms of age distribution, most cases were of age ≤ 34 years in the case (48.3%) and control groups (49.9%). The distributions of comorbidities in the case group, that is, T2DM, sleep disorder, CAD, hypertension, and CKD, were higher than those in the control group. Most patients with schizophrenia received FGAs (82.3%), and 46.1% received SGAs as treatment. There was a statistically significant difference between the T2DM and CAD groups (*p* < 0.05). As shown in Figure 1, the Kaplan–Meier curves for the cumulative risk of hyperlipidemia in the schizophrenia group were significantly higher than those in the non-schizophrenia group (log-rank test, *p* < 0.001).

**Table 1:** Demographic characteristics, comorbidity, and medication in patients with and without schizophrenia.

Variable	Schizophrenia		*P* value
	No	Yes	
	*N* = 4,533	*N* = 4,533	
Sex	*N* (%)	*N* (%)	0.99
Female	2,167 (47.8)	2,167 (47.8)	
Male	2,366 (52.2)	2,366 (52.2)	
Age, mean (SD)	37.7 (13.0)	38.0 (12.4)	0.30
Stratify age			0.13
≤ 34	2,260 (49.9)	2,190 (48.3)	
35–49	1,525 (33.6)	1,617 (35.7)	
50+	748 (16.5)	726 (16.0)	
Comorbidity			
T2DM	99 (2.18)	188 (4.15)	< 0.001
Sleep disorder	8 (0.18)	16 (0.35)	0.10
CAD	168 (3.71)	209 (4.61)	0.03
Hypertension	348 (7.68)	398 (8.78)	0.06
CKD	36 (0.79)	43 (0.95)	0.43
Medication			
FGAs		3,732 (82.3)	
SGAs		2088 (46.1)	

Chi-Square Test; ^#^Two sample *T*-test.

Table 2 summarizes the stratified analysis by sex, age, and comorbidity between patients with and without schizophrenia. Compared to the non-schizophrenia group, patients had a 1.30-fold higher risk of hyperlipidemia (aHR, 1.30; 95% CI, 1.19–1.43) after adjusting for age, sex, and comorbidity. Schizophrenia patients, males (aHR, 1.33; 95% CI, 1.17–1.51) and females (aHR, 1.28; 95% CI, 1.12–1.46) had a significantly higher risk of developing hyperlipidemia, and patients without schizophrenia were selected as the reference. Patients with schizophrenia within the age group of ≤34 years had a 2.43-fold risk of hyperlipidemia (aHR, 2.43; 95% CI, 2.05–2.87); we selected patients without schizophrenia in the ≤34 years group as reference. When patients with schizophrenia without other comorbidities (i.e., T2DM, sleep disorder, CAD, hypertension, and CKD) had a higher risk of hyperlipidemia (aHR, 1.37; 95% CI, 1.24–1.52), selected patients without schizophrenia and those without comorbidities were used as references.

**Table 2:** Comparison of incidence and hazard ratio of hyperlipidemia stratified by sex, age, and comorbidity between patients with and without schizophrenia.

Variable	Schizophrenia							
	No			Yes				
	Event	PY	Rate^#^	Event	PY	Rate^#^	Crude HR (95% CI)	Adjusted HR^†^ (95% CI)
All	826	44,990	18.4	1,013	41,359	24.5	1.34 (1.22, 1.47)***	1.30 (1.19, 1.43)***
Sex								
Female	390	20,980	18.6	483	19,601	24.6	1.33 (1.16, 1.52)***	1.28 (1.12, 1.46)***
Male	436	24,010	18.2	530	21,758	24.4	1.35 (1.19, 1.53)***	1.33 (1.17, 1.51)***
Stratify age								
≤ 34	202	23,994	8.42	437	20,933	20.9	2.51 (2.12, 2.97)***	2.43 (2.05, 2.87)***
35–49	381	15,217	25.0	415	14,883	27.9	1.12 (0.97, 1.29)	1.07 (0.93, 1.23)
50+	243	5,779	42.1	161	5,543	29.0	0.69 (0.57, 0.84)***	0.69 (0.56, 0.84)***
Comorbidity^‡^								
No	650	41,304	15.7	809	36,879	21.9	1.40 (1.27, 1.56)***	1.37 (1.24, 1.52)***
Yes	176	3,686	47.8	204	4,480	45.5	0.95 (0.77, 1.16)	0.94 (0.77, 1.16)

Rate^#^, incidence rate, per 1,000 person-years; Crude HR, crude hazard ratio. Adjusted HR^†^: multivariable analysis including age, sex, and comorbidities of T2DM, sleep disorder, CAD, hypertension, and CKD. Comorbidity^‡^: Patients with any one of the comorbidities T2DM, sleep disorder, CAD, hypertension, and CKD were classified as the comorbidity group. ***p < 0.001.

Table 3 shows that patients with schizophrenia without APs treatment showed a 2.16-fold (95% CI, 1.79–2.60) increased risk of hyperlipidemia, with selected patients without schizophrenia serving as a reference. We stratified patients with schizophrenia who received different APs treatments based on the use of FGAs, SGAs, or both. Compared with patients with schizophrenia not receiving APs treatment, patients taking FGAs (aHR, 0.55; 95% CI, 0.46–0.67), SGAs (aHR, 0.42; 95% CI, 0.29–0.60), or both treatments (aHR, 0.55; 95% CI, 0.45–0.66) had a low risk of developing hyperlipidemia after adjusting for sex, age, and comorbidities. The association between APs treatment dosage and hyperlipidemia risk was also analyzed. The most significant lower risk of hyperlipidemia was present in schizophrenia patients with APs treatment exposure, more than the third quartile compared with patients without schizophrenia. Similar results were observed for the different APs treatment doses.

**Table 3:** Incidence, crude, and adjusted hazard ratio of hyperlipidemia compared among schizophrenia patients with and without antipsychotic treatment compared to non-schizophrenia controls associated with annual mean defined daily doses.

Variables	*N*	Event	PY	Rate^#^	Crude HR (95% CI)	Adjusted HR^†^ (95% CI)	Adjusted HR^†^ (95% CI)
Non-schizophrenia controls	4,533	836	44,990	18.4	1 (Reference)	1 (Reference)	
Schizophrenia without anti-psychotic treatment	500	131	3,021	43.4	2.41 (2.00, 2.90)***	2.16 (1.79, 2.60)***	1 (Reference)
Schizophrenia with anti-psychotic treatment							
FGAs	3,732	845	36,260	23.3	1.27 (1.15, 1.40)***	1.25 (1.13, 1.38)***	0.55 (0.46, 0.67)***
< 1,150 DDD	2,291	688	19,661	35.0	1.93 (1.74, 2.14)***	1.87 (0.69, 2.07)***	0.81 (0.67, 0.98)*
≥ 1,150 DDD	1,441	157	16,599	9.46	0.51 (0.43, 0.61)***	0.51 (0.43, 0.61)***	0.22 (0.17, 0.28)***
SGAs	296	36	2059	17.5	0.97 (0.70, 1.36)	0.92 (0.66, 1.29)	0.42 (0.29, 0.60)***
< 250 DDD	132	28	888	31.5	1.77 (1.21, 2.58)**	1.65 (1.13, 2.40)**	0.74 (0.49, 1.11)
≥ 250 DDD	164	8	1,171	6.83	0.38 (0.19, 0.77)**	0.35 (0.18, 0.71)**	0.17 (0.08, 0.34)***
Both	4,033	882	38,338	23.0	1.26 (1.14, 1.38)***	1.23 (1.12, 1.36)***	0.55 (0.45, 0.66)***
< 1,600 DDD	2,588	756	21,588	35.0	1.94 (1.76, 2.14)***	1.86 (1.68, 2.05)***	0.80 (0.67, 0.97)*
≥ 1,600 DDD	1,445	126	16,751	7.52	0.41 (0.34, 0.49)***	0.41 (0.34, 0.50)***	0.17 (0.14, 0.22)***

Rate^#^, incidence rate, per 1,000 person-years; Crude HR, crude hazard ratio. Adjusted HR^†^: multivariable analysis including age, sex, and comorbidities of T2DM, sleep disorder, CAD, hypertension, and CKD. *p < 0.05; **p < 0.01; ***p < 0.001.

### Effects of APs on lipid catabolism-related gene expression

**Figure 2:**
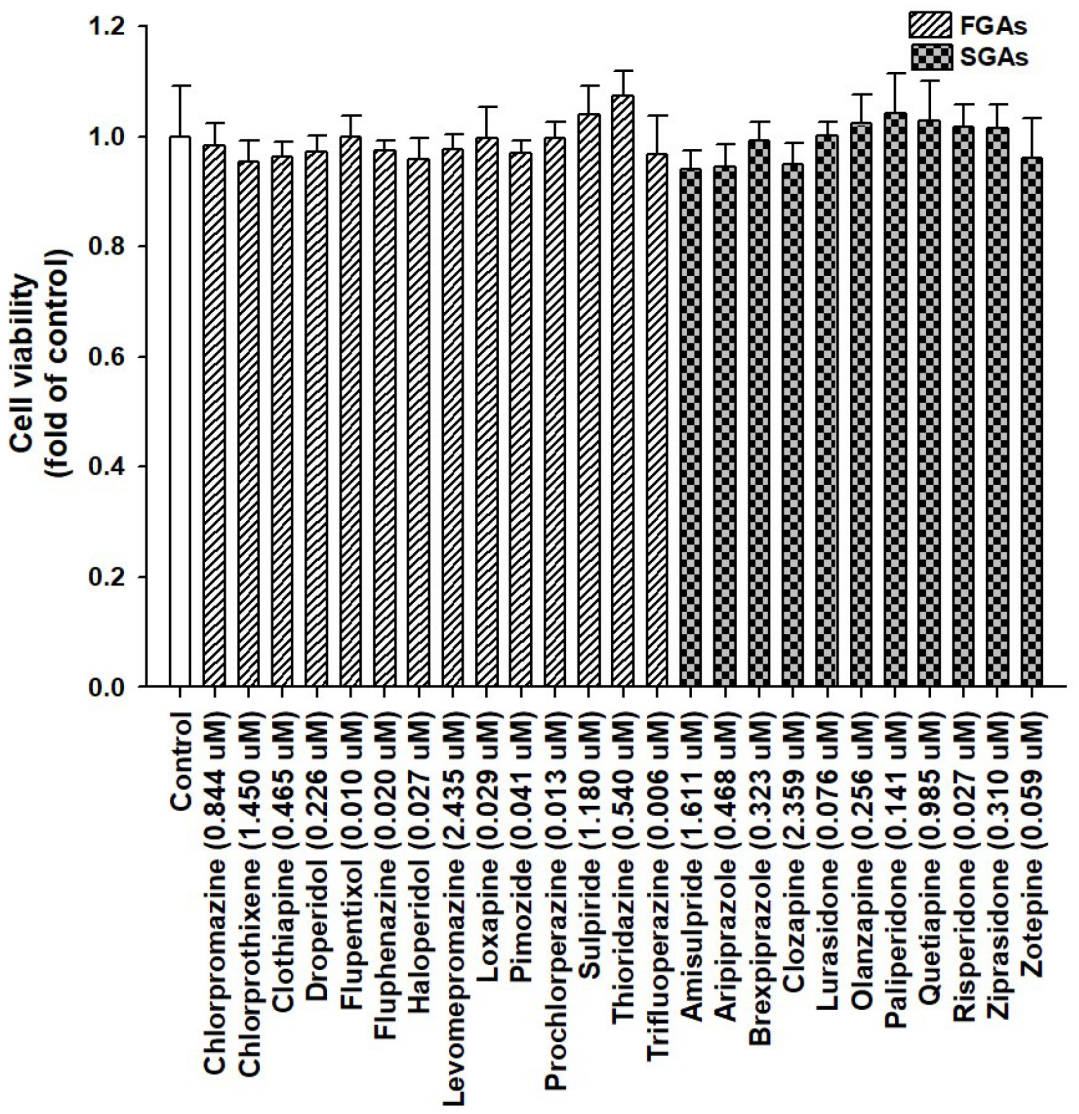
Viability of HepaRG cells following exposure to antipsychotic medications (APs). HepaRG cells were exposed to FGAs [Chlorpromazine (0.844 μM), Chlorprothixene (1.450 μM), Clothiapine (0.465 μM), Droperidol (0.226 μM), Flupentixol (0.010 μM), Fluphenazine (0.020 μM), Haloperidol (0.027 μM), Levomepromazine (2.435 μM), Loxapine (0.029 μM), Pimozide (0.041 μM), Prochlorperazine (0.013 μM), Sulpiride (1.180 μM), Thioridazine (0.540 μM), and Trifluoperazine (0.006 μM)] and SGAs [Amisulpride (1.611 μM), Aripiprazole (0.468 μM), Brexpiprazole (0.323 μM), Clozapine (2.359 μM), Lurasidone (0.076 μM), Olanzapine (0.256 μM), Paliperidone (0.141 μM), Quetiapine (0.985 μM), Risperidone (0.027 μM), Ziprasidone (0.310 μM), and Zotepine (0.059 μM)] for 72 h. Cell viability was monitored by measuring cellular acid phosphatase activity using *p*-nitrophenylphosphate as a substrate. The data shown are the mean ± standard error (SE) (*n* = 3).

We used APs available in Taiwan, such as FGAs (chlorpromazine, chlorprothixene, clothiapine, droperidol, flupentixol, fluphenazine, haloperidol, levomepromazine, loxapine, pimozide, prochlorperazine, sulpiride, thioridazine, and trifluoperazine) and SGAs (amisulpride, aripiprazole, brexpiprazole, clozapine, lurasidone, olanzapine, paliperidone, quetiapine, risperidone, ziprasidone, and zotepine). Based on the highest possible plasma or serum drug concentrations, the concentrations were chosen (30–38). In a prior investigation, the liver toxicity of these medications was documented and reviewed (39). Therefore, using HepaRG cells to carry out a cell viability assay, we evaluated the toxicity of these APs. The concentrations of FGAs used for the study were 0.844, 1.450, 0.465, 0.226, 0.010, 0.020, 0.027, 2.435, 0.029, 0.041, 0.013, 1.180, 0.540, and 0.006 μM (for chlorpromazine, chlorprothixene, clothiapine, droperidol, flupentixol, fluphenazine, haloperidol, levomepromazine, loxapine, pimozide, prochlorperazine, sulpiride, thioridazine, trifluoperazine, respectively). The concentrations of SGAs used for the study were 1.611, 0.468, 0.323, 2.359, 0.076, 0.256, 0.141, 0.985, 0.027, 0.310, and 0.059 μM (for amisulpride, aripiprazole, brexpiprazole, clozapine, lurasidone, olanzapine, paliperidone, quetiapine, risperidone, ziprasidone, zotepine, respectively). The cells were treated with these APs at these concentrations for 72 h, and the ACP assay was performed to assess cell viability. Our results showed no significant differences between the control and APs-treated cells (Figure 2).

**Figure 3:**
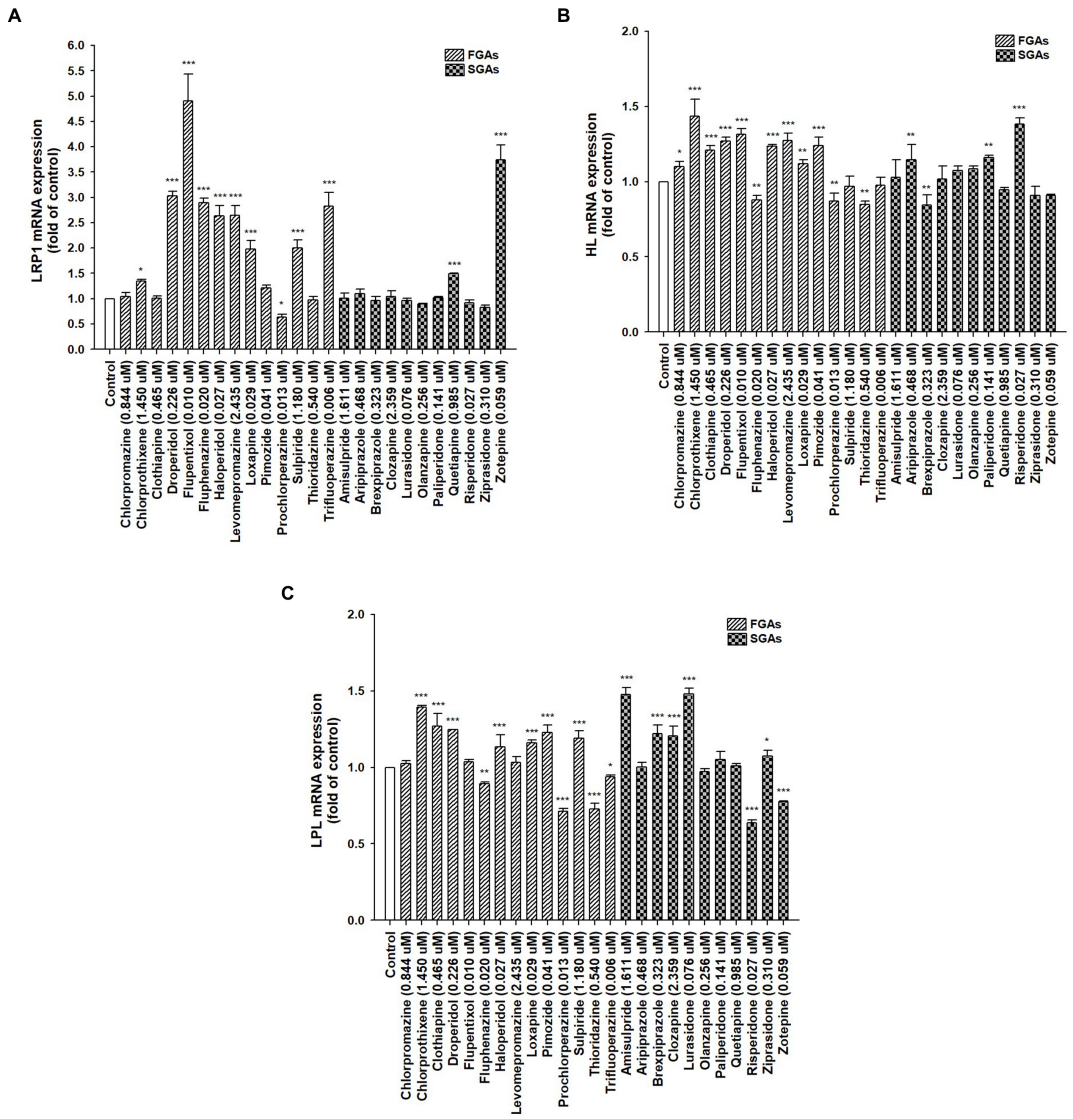
Expression of hepatic lipid homeostasis-related genes following treatment with antipsychotic medications (APs). Differentiated HepaRG cells were treated for 72 h with FGAs [Chlorpromazine (0.844 μM), Chlorprothixene (1.450 μM), Clothiapine (0.465 μM), Droperidol (0.226 μM), Flupentixol (0.010 μM), Fluphenazine (0.020 μM), Haloperidol (0.027 μM), Levomepromazine (2.435 μM), Loxapine (0.029 μM), Pimozide (0.041 μM), Prochlorperazine (0.013 μM), Sulpiride (1.180 μM), Thioridazine (0.540 μM), and Trifluoperazine (0.006 μM)] and SGAs [Amisulpride (1.611 μM), Aripiprazole (0.468 μM), Brexpiprazole (0.323 μM), Clozapine (2.359 μM), Lurasidone (0.076 μM), Olanzapine (0.256 μM), Paliperidone (0.141 μM), Quetiapine (0.985 μM), Risperidone (0.027 μM), Ziprasidone (0.310 μM), and Zotepine (0.059 μM)]. Following treatment, RNA was extracted, and the expression levels of **(A)** *LRP1*; **(B)** *HL*; and **(C)** *LPL* were analyzed by quantitative reverse transcription-polymerase chain reaction. Values were normalized to the expression of *β-actin*, with the *β-actin* levels of dimethyl sulfoxide (DMSO)-treated cells set at 1. Results are expressed as means ± standard error (SE) (*n* = 3), *, *p* < 0.05; **, *p* < 0.01; ***, *p* < 0.001 compared with cells treated with DMSO. LRP1, low-density lipoprotein receptor-related protein 1; HL, hepatic lipase; LPL, lipoprotein lipase.

Lipid catabolism-related genes, *LRP1*, *HL*, and *LPL* (Figure 3), and lipogenesis-related genes, *FAS*, *SCD*, *SREBP-1c*, and *LXRα* (Supplementary Figure S1) were assessed by real-time PCR in differentiated HepaRG cells to determine the expression of hepatic genes associated with lipid catabolism. The aforementioned APs were applied to differentiated HepaRG cells for 72 h. Total RNA was harvested, and the expression of the indicated genes was analyzed. Among the 14 FGAs, 64.3, 64.3, and 50% induced *LRP1*, *HL*, and *LPL* expression, respectively. Of the 11 SGAs, 18.2, 27.3, and 45.5% significantly induced *LRP1*, *HL*, and *LPL* expression, respectively. FGAs appeared to have a more prominent inducing effect on these genes than SGAs did. Although most drugs decreased lipogenesis-related gene expression, some increased the expression of these genes (not more than 2-fold; Supplementary Figure S1), suggesting that lipogenesis may play a minor role in AP-treated cells rather than lipid catabolism-related genes. These results suggest that AP treatment in patients with schizophrenia reduces hyperlipidemia and may partly induce hepatic gene expression involved in lipid catabolism, thus leading to improved blood lipid profiles.

## Discussion

Comprehensive research on the effects of applying APs and the risk of developing hyperlipidemia in people with schizophrenia, particularly in the Asian community, is lacking. So, utilizing the NHIRD, we provide a study on the occurrence of hyperlipidemia in schizophrenia patients and the use of APs in treating schizophrenia in Asian adults. After controlling for several significant and associated variables, we discovered that the risk of hyperlipidemia in patients with schizophrenia was 1.30 times higher than that of the general population in Taiwan (*p* < 0.001). Our study contains various noteworthy aspects, including: (1) This distinctive countrywide, population-based cohort study offers a thorough and in-depth observation of an Asian population and finds a statistically significant correlation between the incidence of developing hyperlipidemia and patients with schizophrenia; (2) without APs treatment, patients with schizophrenia had a significantly higher incidence of hyperlipidemia than patients in the control group (aHR, 2.16; 95% CI, 1.79–2.60, *p* < 0.001); (3) with APs treatment, patients with schizophrenia had a significantly lower risk of developing hyperlipidemia than those who did not receive APs (all aHR ≤ 0.55); (4) using hepatocyte-derived HepaRG cells, we treated various APs and discovered that most of them, particularly FGAs, significantly increased the expression of crucial lipid catabolism genes, including LRP1, HL, and LPL. This may help explain why blood lipid profiles are now better. With improved blood lipids, a better prognosis, and a lower risk of developing CVD, this study may aid doctors in treating schizophrenia patients and better understanding the impact of APs.

The risk of CVD-related premature death is much higher in those with schizophrenia. The relationship between schizophrenia and CVD has been proposed to be mediated by MetS, including hypertension, diabetes, and hyperlipidemia. Hyperlipidemia, the most dangerous risk factor, leads to CVD, stroke, and related cardiovascular defects. The role of hyperlipidemia in CVD development has been reported previously. With an increase of TG to 88.5 mg/dL (1 mmol/L), the risk of CVD increased by 12 and 37% in men and women, respectively. In addition, in a meta-analysis, each 38.7 mg/dL (1 mmol/L) drop in LDL-C reduced the risk of CVD by 23% (40). It is vital to manage AP-induced hyperlipidemia in patients with schizophrenia to reduce the risk of CVD. Our results are consistent with a study by Mitchell et al. (41), wherein patients with schizophrenia had a higher prevalence and incidence of hyperlipidemia than the general population. Several factors should be considered when developing treatment regimens for patients with schizophrenia. First, the exact features of schizophrenia-associated illnesses should be examined. Schizophrenia is thought to be brought on by several endogenous factors, including endocrine state, neurohormonal imbalance, and autonomic nerve imbalance. So, one of the independent risk factors underlying the risk of hyperlipidemia is schizophrenia itself. Patients with schizophrenia often maintain sedentary lifestyles, eat unhealthily, do not exercise, and smoke. Medication side effects and genetic predispositions can also contribute to the development of hyperlipidemia in these patients. One or more of these metabolic risk factors may be present in many drug-naïve patients. Genes associated with metabolic function intersect with biological and genetic components of schizophrenia and treatment outcomes. Independent research has linked the genes that govern inflammation, glucose metabolism, leptin signaling, and fat mass to schizophrenia (2).

Our results showed that patients with schizophrenia were more likely to have T2DM and CAD than the general population. The course and outcome of schizophrenia are reportedly heterogeneous since the relationship between blood lipid contents, lipid metabolism, and clinical characteristics of the disease itself and/or current symptoms remains largely unknown. The associations between lipid levels and disease severity may represent fluctuations in lipid levels as the disease progresses with inconsistent results (42, 43). Redox regulation, immunological, and inflammatory pathways and their integration are all part of schizophrenia’s pathogenesis. The role of oxidative stress has also been proposed, which may influence lipid levels (44, 45). Solberg et al. (46) reported higher levels of TG in these patients both at an acute and at a 5-year follow-up period. In a longitudinal study, hyperlipidemia in these patients was primarily studied as an adverse effect of APs treatment (47). Thereafter, APs have been associated with weight gain, which may contribute to blood lipid abnormalities (48). Mechanisms have also been proposed to explain the weight-gain propensity of APs. However, the range of weight gain varied among these APs and patient characteristics. Clozapine and olanzapine are reportedly associated with the highest weight gain (6). It might be related to how they affect peripheral and central nervous system (CNS) muscarinic M_3_ receptors, serotonin 5-HT_2A_ and 5-HT_2C_ receptors, dopamine D_2_ and D_3_ receptors, and histamine H_1_ receptors; their actions trigger the dopamine reward system, causing abnormal signaling along the hypothalamic–pituitary–adrenal axis, and subsequently increasing patients’ appetite. Proopiomelanocortin (POMC), an appetite-inhibiting neuropeptide, is decreased by increased levels of appetite-stimulating neuropeptides such as neuropeptide Y (NPY) and agouti-related peptide (AgRP), which reduces energy expenditure, increases food intake, weight gain, and hyperlipidemia (49). Thus, hyperlipidemia may be a secondary response to AP-induced weight gain. The dopamine, histamine, and serotonin systems, among others, are shared by many metabolically important tissues in the CNS and peripheral tissues. Adipose tissues, the gastrointestinal (GI), liver, muscle, and pancreas are among the target organs in the peripheral that are affected by changes in the central brain’s metabolic regulation, and these tissues respond reciprocally. Due to the simultaneous action of APs on various receptor systems in all of these tissues, the effects of these medications’ disturbances of hunger and metabolic regulation are probably cumulative and result in the metabolic imbalances seen in clinical settings (50). Many researchers have focused on changes in dopamine and serotonin neurotransmitters and receptors, which may originate from the pathogenesis of schizophrenia or APs (51). Changes in the expression of certain genes may play an important role; however, the effects of APs on the expression of these lipid catabolic genes remain unclear, and understanding this phenomenon has important clinical significance in the prevention and treatment of hyperlipidemia. Therefore, the impact of these APs on lipid catabolic gene expression is worth exploring further.

Due to SGAs’ strong affinity for serotonin 5-HT_2C_ receptors, such as clozapine and olanzapine, there is a substantial risk of metabolic side effects (6). Patients who take risperidone and olanzapine have a higher chance of getting MetS than those who take FGAs (52). Numerous studies have estimated the frequency of MetS in individuals receiving APs to range from 15 to 69% (53). A study also found a significant increase in BMI and fasting glucose levels after the first exposure to APs for 9 months; however, there were no significant changes in blood lipid levels (54). SGAs were associated with higher metabolic risk than FGAs; this hints at a differential metabolic liability (i.e., olanzapine ≈ clozapine > risperidone > quetiapine > amisulpride > ziprasidone ≈ lurasidone ≈ aripiprazole) (2). Li et al. (40) investigated how olanzapine affected individuals with schizophrenia’s lipid profiles through a meta-analysis of 21 trials. After one month of treatment, they observed an increase in TG, TC, and LDL-C but no appreciable changes in HDL-C. However, this finding could be challenged because this study did not discuss long-term diet control. Our lipid catabolism gene expression data suggest that olanzapine does not induce lipid catabolism gene expression compared to the untreated group, suggesting that this may be one of the reasons underlying drug-induced hyperlipidemia. According to a previous report, switching to aripiprazole, amisulpride, ziprasidone, or lurasidone may be an alternative treatment for patients with hyperlipidemia, improving their lipid profiles while continuing to treat their mental symptoms (55). However, it is also necessary to evaluate each patient’s condition individually.

Despite the favorable evidence, these studies have several limitations; for example, most studies involved small sample sizes and medicated patients but did not include the lipid profiles of patients who have never been exposed to APs. These studies were largely cross-sectional and have shown a significant variation in prevalence rates among various countries depending on using different definitions (53). Only a few studies have focused on the risk of hyperlipidemia in drug-naïve patients and followed up on these patients longitudinally to assess the possibility of hyperlipidemia development. It is important to note that the cumulative long-term effects of poor health behaviors and more prolonged illness duration may increase the risk of these metabolic disorders. Further extensive studies are required to validate these findings. These discrepancies between our results and prior studies may be due to the sample size, ethnicity, clinical traits of the study populations’ chosen populations, data interpretation, consideration of methodological differences, sampling duration, and baseline characteristics of study samples.

Age independently determines the prevalence of MetS; increasing age is associated with an increased prevalence of MetS in patients with schizophrenia compared with healthy controls (56). However, we showed that with an increase in age, the risk of hyperlipidemia decreased, even in patients receiving APs. On the other hand, a Chinese population study reported consistent results suggesting that age was negatively linked with TC and LDL-C levels in men and women, respectively, in ≥61-year-old groups. Women’s TC, TG, and LDL-C levels were lower than those of men but eventually caught up to men’s levels in the 51–55-, 61–65-, and 51–55-year-old age groups (57). A biological system’s ability to function declines over time as part of the fundamental and natural process of aging. Through intricate mechanisms, aging causes changes in fat and lipoprotein absorption, production, and metabolism. The results show lifetime changes in lipid levels, which may be an indication of how the aging process affects lipid metabolism in the Chinese population as a whole (57). We did not perform lipid profile testing in patients with schizophrenia as a routine test in Taiwan. Thus, the management of hyperlipidemia in these patients and the general population is lacking in our clinical setting. Doctors should evaluate hyperlipidemia in patients with schizophrenia via routine blood lipid profile evaluation and thus provide complete medical care and appropriate treatment.

Based on our findings, we recommend that all patients with schizophrenia must undergo routine metabolic profile monitoring to assess their serum lipid profiles, which provides essential information on CVD risk. We also found that most APs contained induction potential for lipid catabolism gene expression, and these properties may limit the metabolic liabilities that may be preferentially used. When lifestyle modifications and switching APs affect metabolic status, clinicians may consider using agents that are beneficial in terms of managing blood lipids in these patients. Since patients with schizophrenia have a high prevalence of abnormal blood lipid profiles, they should be counseled for lifestyle improvement, and their lipid parameters should be reexamined to improve and monitor their health status. We contend that some drugs may significantly contribute to metabolic dysfunction and that patients should be switched to drugs with lower metabolic impact. Our lipid catabolism gene expression data suggest that which APs induce at least one of these genes and may improve blood lipid levels in patients with schizophrenia. There is also a chance that the psychotic symptoms will return or get worse. Therefore, factors including inadequate psychotic symptom control, side effects, and the potential for hyperlipidemia after switching to other medications should be considered, and the risks and benefits should be weighed before switching.

LRP1 is a critical factor involved in lipid homeostasis. By interacting with ApoE, LPL, and HL, LRP1 facilitates the endocytotic import of food lipids contained in postprandial chylomicron remnants into hepatocytes (24). It reportedly plays a critical role in MetS and has a potential diagnostic value to monitor the development of atherosclerosis and CVD. Loss of LRP1 in the liver may lead to metabolic dysregulation and hepatic steatosis. In a mouse model, LRP1−/− mice displayed elevated TC and TGs levels resulting from the accumulation of large, TG-rich lipoprotein particles in the circulation (58). Another possibility for exploiting LRP1 in MetS could be the potential therapeutic use in terms of developing molecular mediators for inducing LRP1 gene expression (24). A new therapeutic strategy using an artificial LRP1-binding peptide with identical residue sequences as ligands recognizing the specific binding domains of the LRP1 α-subunit to suppress the development of MetS was developed (59). This suggests that developing advanced therapies using LRP1 as a molecular and cellular target for MetS treatment is possible. Among APs, FGAs (arranged according to the inductive ability: flupentixol > droperidol > fluphenazine ≈ trifluoperazine > haloperidol ≈ levomepromazine > loxapine ≈ sulpiride > chlorprothixene) and SGAs (zotepine > quetiapine) had the potential to induce LRP1 expression and thus rescue the patients from the risk of hyperlipidemia. Low HL activity and gene expression are associated with an increased risk of CVD (60). Convincing evidence shows that the anti- or pro-atherogenic role of HL plays a crucial role in remanent lipoprotein catabolism, as well as in the remodeling of LDL-C and HDL-C particles. Overexpression of HL alters lipid profiles in some animal models by reducing cholesterol in apoB-containing lipoproteins and aortic cholesterol content in cholesterol-fed mice (61, 62). Hypertriglyceridemia or hypercholesterolemia is common in HL-deficient patients, who also tend to collect VLDL-C, chylomicron remnants, and LDL-C levels (63). HL also facilitates the uptake of chylomicrons, chylomicron remnants, VLDL-C, and HDL-C via LRP1 into various cell types and protects against aortic atherosclerosis (63). LPL mediates intravascular lipolysis of TG-rich lipoproteins. Hepatic LPL overexpression reduces lipid storage in the liver, hydrolyzes TG to produce FAs, and boosts FA oxidation (26). Chylomicrons, VLDL-C, and LDL-C build up in the plasma due to impaired LPL activity or expression, resulting in hypertriglyceridemia. Hypertriglyceridemia is allegedly an independent risk factor for CVD and a residual risk factor for the development of atherosclerosis, even if LDL-C is acknowledged as a primary risk factor for atherosclerosis (64, 65). We found that, among APs, more FGAs induced HL and LPL gene expression compared to SGAs.

We also tested whether these drugs affected lipogenesis-related gene expression (FAS, SCD, SREBP-1c, and LXRα) (66) (Supplementary Figure S1). We found that, although most drugs decreased gene expression, some increased the expression of these genes (not more than 2-fold), suggesting that lipogenesis may play a minor role in APs-treated cells rather than lipid catabolism-related genes. These dual effects of APs, a direct increase in lipid catabolism gene expression, reduced hyperlipidemia risk, and control disease-illness status, might benefit patients with schizophrenia. However, it may not be evident how APs may ultimately affect lipid profiles in terms of CVD risk. Furthermore, it is crucial to remember that APs would probably only be helpful in patients with schizophrenia and not in the general population when considering the consequences of AP use on the benefit of blood lipid profile.

Our study used a longitudinal population-based cohort to examine the epidemiology of hyperlipidemia risk in patients with schizophrenia and the general control cohort in Taiwan. Cases and controls were matched for age and sex. We also assessed the potential for hyperlipidemia among patients on APs for schizophrenia. We further analyzed lipid homeostasis-related gene expression in hepatic cells following APs treatment. However, this study has several limitations: (1) Misclassification is possible in this claims insurance database. Thus it is important to consider the quality and dependability of the secondary data on schizophrenia and hyperlipidemia. To combat false positives, a peer-review system has been formed in Taiwan, where doctors are paid by administrative specialists, and universal health insurance has been implemented. (2) A few unmeasured confounding risk variables for hyperlipidemia might not have been included in the study. For instance, access to patient personal information, including smoking, alcohol use, BMI, natural presentation, work position, lifestyle habits, dietary intake, and family history of hyperlipidemia, was unavailable through the NHIRD. (3) The NHIRD claims database was only created for billing purposes. As a result, some data was anonymized, and even after contacting the patients directly, we could not retrieve their specific patient data. (4) This study did not address the severity or evolution of hyperlipidemia in patients with schizophrenia. (5) Different study designs and statistical and methodological limitations of this study could make its comparison with other studies difficult. (6) Since NHIRD lacks specialized research facilities, we could not assess the degree of hyperlipidemia in patients with schizophrenia, including TC, TGs, HDL-C, and LDL-C. As a result, our case definition was based on diagnoses that doctors documented in accordance with patients’ post-APs lipid levels. In this investigation, professionals accurately assessed and categorized both schizophrenia and hyperlipidemia in accordance with accepted clinical criteria, taking into account typical side effects, symptoms, and research facility details. As a result, accounting for comorbidities in our analysis reduced the confounding effects of medications. Similar to other nations, schizophrenia is underdiagnosed in Taiwan, or at least under-recorded; but, unlike other nations, psychiatrists in Taiwan do not frequently prescribe APs unless the diagnosis of schizophrenia is confirmed. As a result, the information on APs in our study is trustworthy and strongly correlated with clinical diagnoses of schizophrenia. In order to undertake a prospective study or randomized controlled trial to examine the association between schizophrenia, APs, and hyperlipidemia in the future, more information must be gathered from other databases or by administering questionnaires. The most important factor in ensuring that patients with schizophrenia receive the best care possible is probably raising doctors’ awareness of the illness and igniting their interest in it through education. The fact that this study’s conclusions were based on a population-based database with a countrywide sample size may help to reduce selection bias in large-scale analyses. To the best of our knowledge, this is also the first study to look into the relationship between AP exposure and the risk of hyperlipidemia in patients with schizophrenia as well as the impact of APs on genes involved in lipid homeostasis.

According to our findings, schizophrenia history is linked to the development of hyperlipidemia, and using the drug lowers the chance of hyperlipidemia later developing. We further verified that, in a hepatic cell line model, AP treatment boosted the expression of genes related to lipid catabolism while decreasing the expression of genes associated with lipogenesis. The positive effects on blood lipid profiles may result from this. However, we believe that more big population-based research or large-scale randomized clinical trials are required to confirm our finding that these APs play a role in preventing hyperlipidemia before any firm conclusions can be drawn. Additionally, it is strongly advised to discover metabolic problems early, monitor them, treat them aggressively and completely, and correct acquired risk factors. We demonstrated the *in vitro* effects of APs on lipid homeostasis gene expression; however, the biological and psychosocial mechanisms underlying these findings warrant further investigation.

## Conclusion

Our study concludes that AP treatment reduces the risk of hyperlipidemia in patients with schizophrenia. Most APs, especially FGAs, stimulate lipid catabolism gene expression, not mainly through inhibiting the lipogenesis pathway. Although most previous studies showed that APs-induced potential metabolic side effects, we confirmed that these side effects did not directly act on lipid homeostasis-related genes but may be on the dopamine reward system, leading to metabolic burden. In addition, schizophrenia patients’ “illness-related” pathophysiological vulnerability to metabolic issues has been linked to their unhealthy lifestyle, lower likelihood of exercising, as well as a potential genetic predisposition. Clinicians who prescribe APs and treat patients taking them need to be aware of the different APs’ metabolic liabilities. Clinical professionals prescribing APs would be in charge of screening for and monitoring metabolic adverse effects. When initiating APs more rigorously with the onset of metabolic disorders, treatments for a healthy lifestyle must be made available to all patients. The only pharmacological strategy with a track record of success in addressing metabolic issues is switching the APs to one with a relatively low metabolic liability. In order to manage patients with major mental diseases and improve their overall results, case management and a multidisciplinary and collaborative care strategy would be helpful.

## Data availability statement

The original contributions presented in the study are included in the article/Supplementary material, further inquiries can be directed to the corresponding authors.

## Ethics statement

The studies involving human participants were reviewed and approved by Institutional Review Board of China Medical University Hospital Research Ethics Committee [CMUH109-REC2-031(CR-2)]. Written informed consent for participation was not required for this study in accordance with the national legislation and the institutional requirements.

## Author contributions

T-YW, Y-JF, and Y-PL: conceptualization. NT, Y-JF, and Y-PL: methodology. C-LL, Y-CC, and CH: software. T-YW and Y-PL: validation. C-LL, Y-CC, CH, and F-JT: formal analysis. T-YW, Y-JF, and Y-PL: investigation. C-LL, CH, F-JT, and Y-PL: resources. C-LL, Y-CC, and Y-PL: data curation. T-YW, NT, Y-JF, and Y-PL: supervision and funding acquisition. CH and Y-PL: project administration. T-YW, NT, C-LL, Y-CC, CH, F-JT, Y-JF, and Y-PL: writing—original draft preparation, writing—review and editing, and visualization. All authors contributed to the article and approved the submitted version.

## Funding

The Taichung Tzu Chi Hospital, Buddhist Tzu Chi Medical Foundation (TTCRD112-28), Show Chwan Memorial Hospital, Changhua, Taiwan (SRD-111024), Taiwan Ministry of Health and Welfare Clinical Trial Center (MOHW110-TDU-B-212-124004), China Medical University (CMU111-MF-34), and the Ministry of Science and Technology, Taiwan, R.O.C. (MOST110-2320-B-039-016-MY3) all provided financial support for this study. We are appreciative of the administrative, technical, and financial support provided by the Health Data Science Center, China Medical University Hospital. The study’s design, data collection, and analysis, publication choice, and article preparation were all done independently from the funders. For this investigation, no extra outside funding was provided.

## Conflict of interest

The authors declare that the research was conducted in the absence of any commercial or financial relationships that could be construed as a potential conflict of interest.

## Publisher’s note

All claims expressed in this article are solely those of the authors and do not necessarily represent those of their affiliated organizations, or those of the publisher, the editors and the reviewers. Any product that may be evaluated in this article, or claim that may be made by its manufacturer, is not guaranteed or endorsed by the publisher.

## Supplementary material

The Supplementary material for this article can be found online at: https://www.frontiersin.org/articles/10.3389/fmed.2023.1137977/full#supplementary-material

## Figures and Tables

**Table:** 

ACP	Acid phosphatase
ADA	American Diabetes Association
AgRP	Agouti-related peptide
APA	American Psychiatric Association
apoE	Apolipoprotein E
APs	Antipsychotics
CAD	Coronary artery disease
CHD	Coronary heart disease
CI	Confidence interval
CKD	Chronic kidney disease
CNS	Central nervous system
CVD	Cardiovascular disease
DMSO	Dimethyl sulfoxide
FA	Fatty acid
FAS	Fatty acid synthase
FFAs	Free fas
FGAs	First-generation aps
GI	Gastrointestinal
HDL-C	High density lipoprotein cholesterol
HL	Hepatic lipase
HR	Hazard ratio
ICD-9-CM, ICD-10-CM	International Classification of Diseases, Ninth and Tenth Revision, Clinical Modification
KM	Kaplan–Meier
LDL-C	Low density lipoprotein cholesterol
LHID	Longitudinal Health Insurance Database
LPL	Lipoprotein lipase
LRP1	LDL-receptor-related protein 1
LXRs	Liver X receptors
MetS	Metabolic syndrome
NHIRD	National Health Insurance Research Database
NHRI	National Health Research Institutes
NPY	Neuropeptide Y
PNPP	Para-nitrophenylphosphate
POMC	Proopiomelanocortin
qRT-PCR	Quantitative real-time polymerase chain reaction
SCD	Stearoyl CoA desaturase
SD	Standard deviation
SE	Standard error
SGAs	Second-generation APs
SREBP-1c	Sterol regulatory element binding protein 1c
T2DM	Type 2 diabetes mellitus
TC	Total cholesterol
TGs	Triglycerides
