# Supplementary material for: Influence of antipsychotic medications on hyperlipidemia risk in patients with schizophrenia: evidence from a population-based cohort study and in vitro hepatic lipid homeostasis gene expression
Source: Front Med (Lausanne). 2023 Jun 22;10:1137977. doi: 10.3389/fmed.2023.1137977 (PMC10324036; doi:10.3389/fmed.2023.1137977)
Supplement: Supplementary file 1 [file Data_Sheet_1.doc]

**Supplemental Table 1.**

**Sequences of PCR primers.**

| **Gene** | **Species** | **Tm (°C)** | **Forward primer (5’-3’)** | **Reverse primer (5’-3’)** |
| --- | --- | --- | --- | --- |
| *LRP1* | human | 60 | ACA TAT AGC CTC CAT CCT AAT C | TTC CAA TCT CCA CGT TCA T |
| *HL* | human | 60 | TAC AGG AGT GCG GCT TCA A | TGC CAG ATC CAG TTT TCT AGC |
| *LPL* | human | 60 | CAG CAG CAA AAC CTT CAT GGT | AGT TTT GGC ACC CAA CTC TCA |
| *LXRα* | human | 60 | CGA TC GAG GTG ATG CTT CTG | GGC AAA GTC TTC CCG GTT AT |
| *SREBP-1c* | human | 60 | CGC TCC TCC ATC AAT GAC AA | TGC AGA AAG CGA ATG TAG TCG AT |
| *FAS* | human | 60 | ACA TCA TCG CTG GTG GTC TG | GGA GCG AGA AGT CAA CAC GA |
| *SCD* | human | 60 | CCG ACG TGG CTT TTT CTT CT | GCG TAC TCC CCT TCT CTT TGA C |
| *β-actin* | human | 60 | CCT GGC ACC CAG CAC AAT | GCC GAT CCA CAC GGA GTA CT |

**Supplemental Figure 1.**

Expression of hepatic lipogenesis-related genes following treatment with antipsychotic medications (APs). Differentiated HepaRG cells were treated for 72 h with FGAs [Chlorpromazine (0.844 μM), Chlorprothixene (1.450 μM), Clothiapine (0.465 μM), Droperidol (0.226 μM), Flupentixol (0.010 μM), Fluphenazine (0.020 μM), Haloperidol (0.027 μM), Levomepromazine (2.435 μM), Loxapine (0.029 μM), Pimozide (0.041 μM), Prochlorperazine (0.013 μM), Sulpiride (1.180 μM), Thioridazine (0.540 μM), and Trifluoperazine (0.006 μM)] and SGAs [Amisulpride (1.611 μM), Aripiprazole (0.468 μM), Brexpiprazole (0.323 μM), Clozapine (2.359 μM), Lurasidone (0.076 μM), Olanzapine (0.256 μM), Paliperidone (0.141 μM), Quetiapine (0.985 μM), Risperidone (0.027 μM), Ziprasidone (0.310 μM), and Zotepine (0.059 μM)]. Following treatment, RNA was extracted, and the expression levels of (A) *LXRα*; (B) *SREBP-1c*; (C) *FAS*; and (D) *SCD* were analyzed by quantitative reverse transcription-polymerase chain reaction. Values were normalized to the expression of *β-actin*, with the *β-actin* levels of dimethyl sulfoxide (DMSO)-treated cells set at 1. Results are expressed as means ± standard error (SE) (n = 3), *, *P* < 0.05; **, *P* < 0.01; ***, *P* < 0.001 compared with cells treated with DMSO. LXRα, liver X receptor alpha; SREBP-1c, sterol regulatory element binding protein 1c; FAS, fatty acid synthase; SCD, stearoyl-CoA desaturase-1.

(B)

(B)

**(A)**


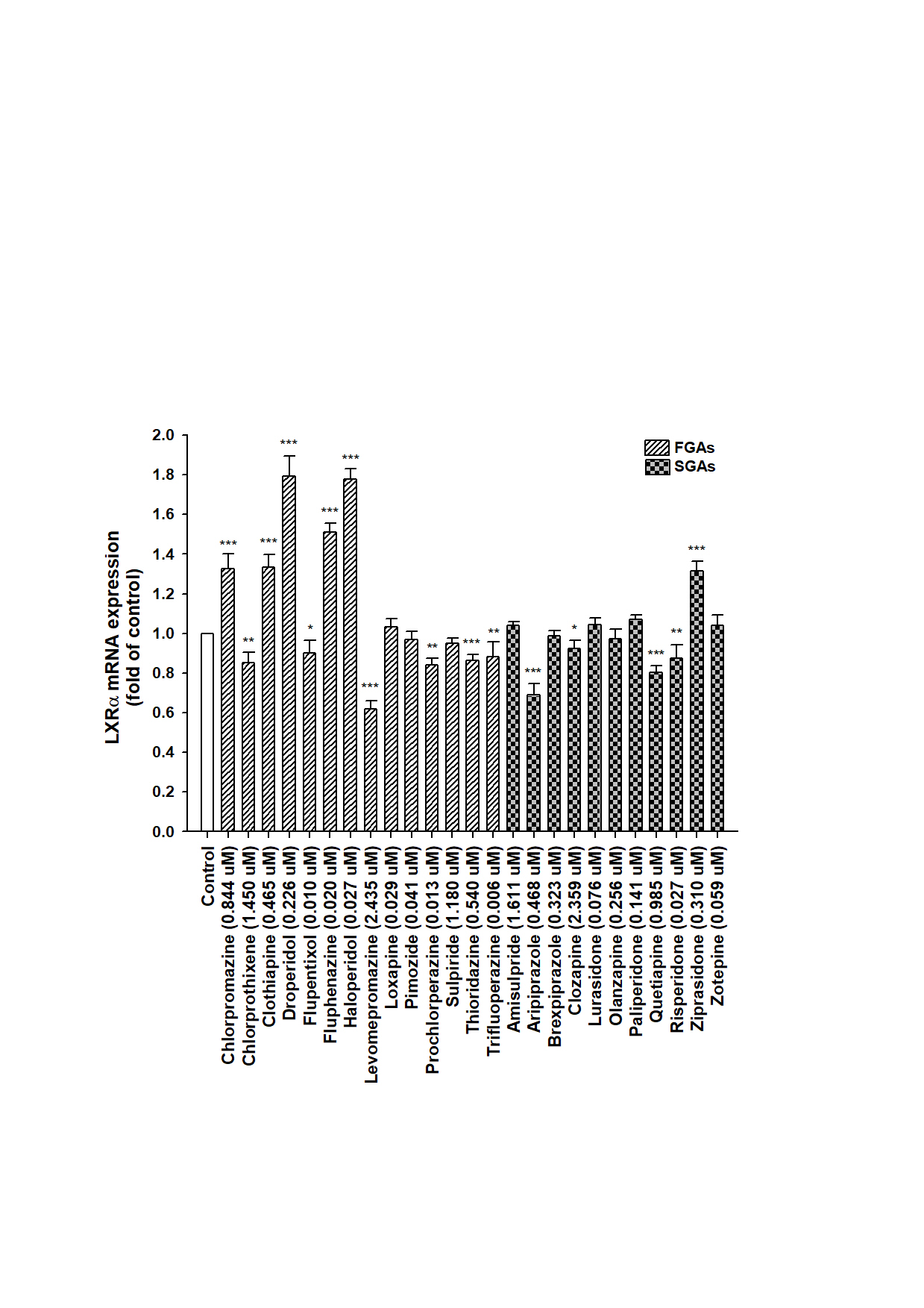


**
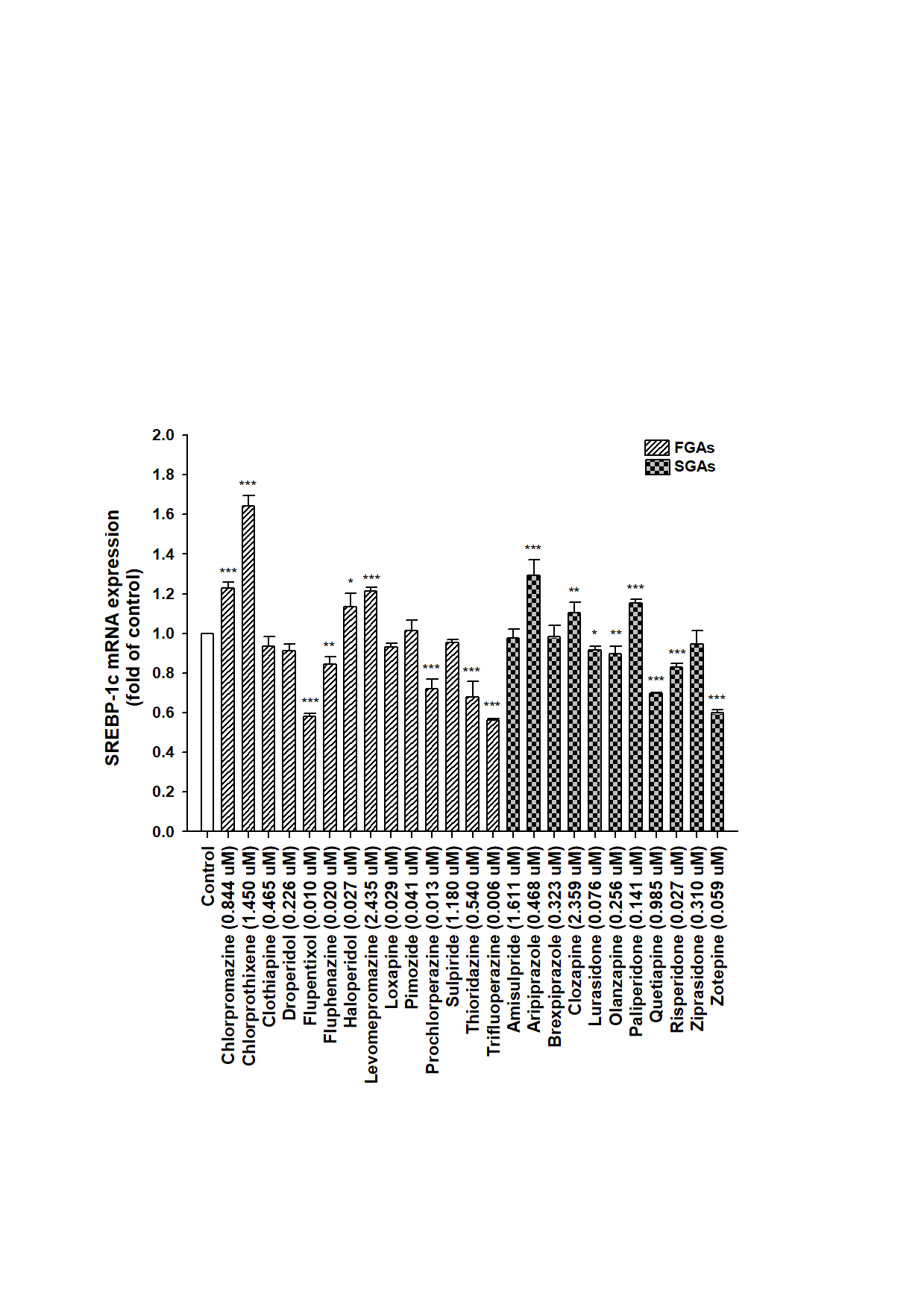
(B)**

**
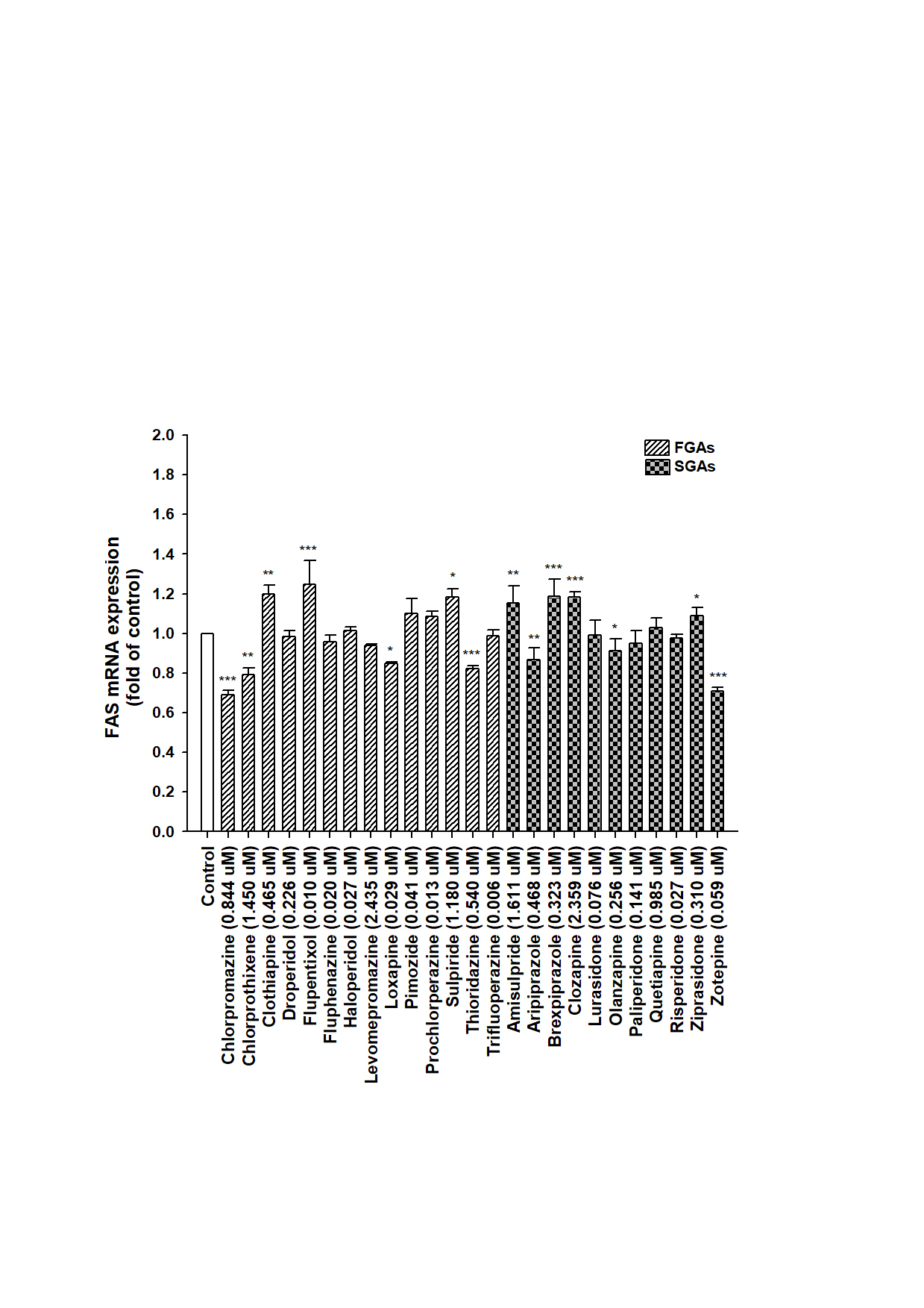
(C)**

**
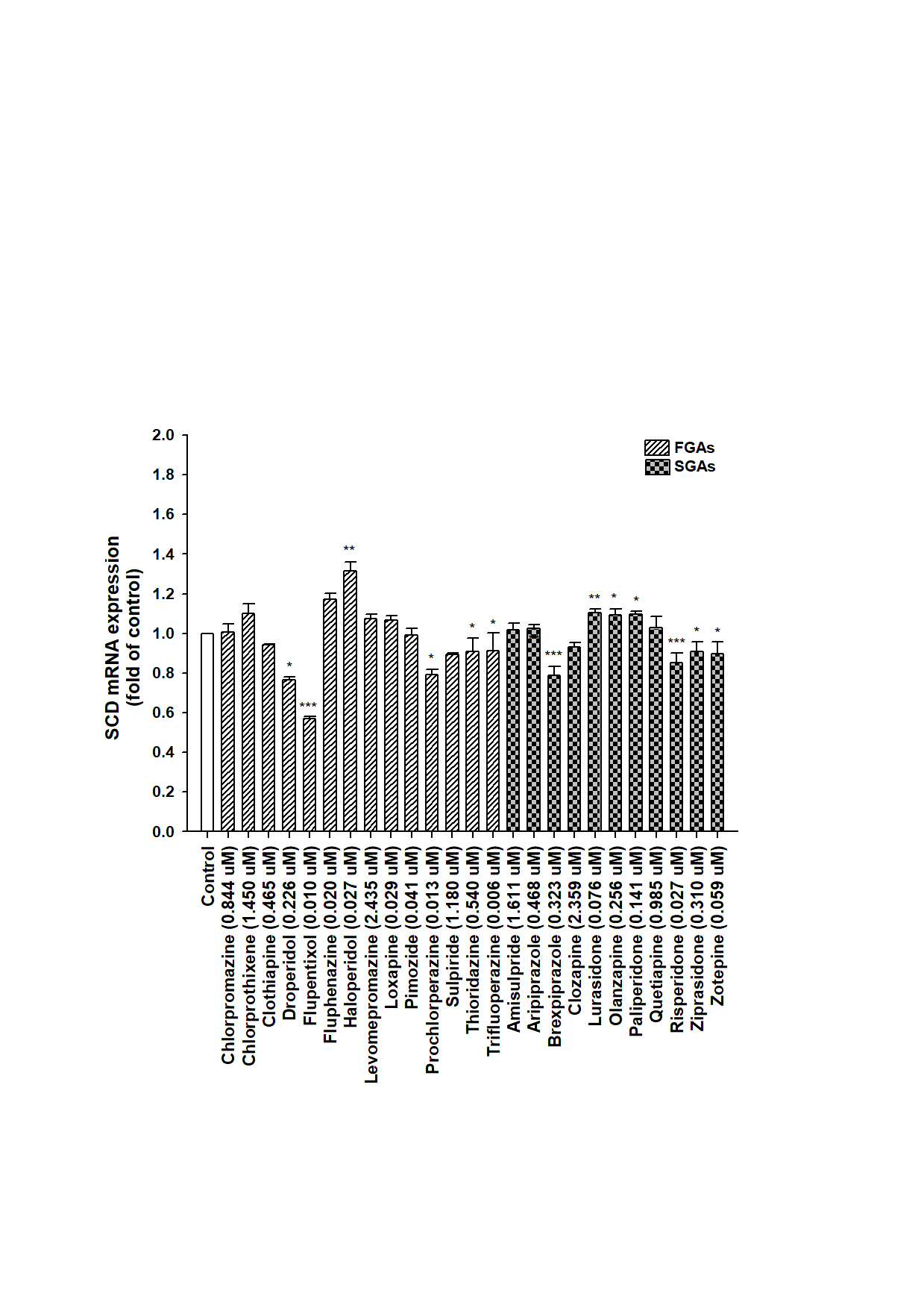
(D)**
